# Celiac disease causing severe osteomalacia: an association still present in Morocco!

**DOI:** 10.11604/pamj.2014.19.43.2757

**Published:** 2014-09-19

**Authors:** Latifa Tahiri, Hamida Azzouzi, Ghita Squalli, Fatimazahra Abourazzak, Taoufik Harzy

**Affiliations:** 1 Department of Rheumatology, CHU Hassan II, Fès, Morocco

**Keywords:** Osteomalacia, celiac disease, vitamin D, bone loss, Morocco

## Abstract

Celiac disease (CD), a malabsorption syndrome caused by hypersensitivity to gliadin fraction of gluten. CD can manifest with classic symptoms; however, significant myopathy and multiple fractures are rarely the predominant presentation of untreated celiac disease. Osteomalacia complicating celiac disease had become more and more rare. We describe here a case of osteomalacia secondary to a longstanding untreated celiac disease. This patient complained about progressive bone and muscular pain, weakness, fractures and skeletal deformities. Radiological and laboratory findings were all in favor of severe osteomalacia. Improvement of patient's weakness and laboratory abnormalities was obvious after treatment with gluten free diet, vitamin D, calcium and iron. This case affirms that chronic untreated celiac disease, can lead to an important bone loss and irreversible complications like skeletal deformities.

## Introduction

Celiac disease (CD) is a chronic digestive disease that results in hypersensitivity to the gliadin fraction of Gluten. Classically, the disease manifests with diarrhea, sometimes steatorrhea, weight loss and complications caused by anemia [1, 2]. There are very few reports of osteomalacia as the presenting symptom, and even fewer of osteomalacia as the only symptom of celiac disease at presentation [2]. Similarly, significant myopathy and multiple fractures are rarely the predominant presentation of untreated celiac disease [3]. In this article, we will present a patient referred to our hospital with an extremely low bone mineral density due to severe osteomalacia with longstanding celiac disease.

## Patient and observation

**Figure 1:**
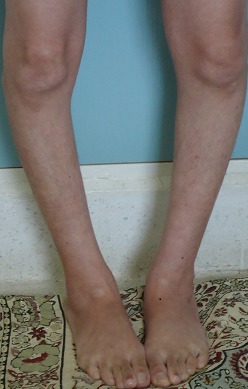
Genu varum

**Figure 2:**
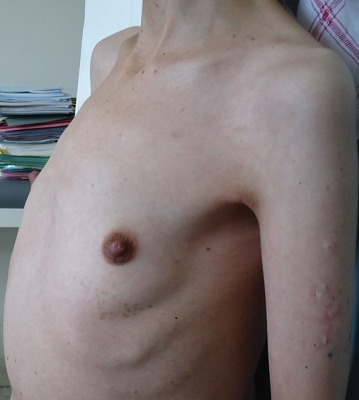
Dermatitis herpetiformis and the hyperbolic chest of the patient

**Figure 3:**
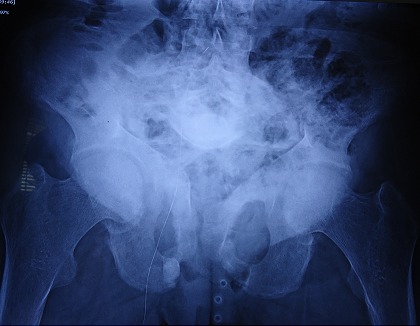
Multiples fractures of the pelvis (arrows)

**Figure 4:**
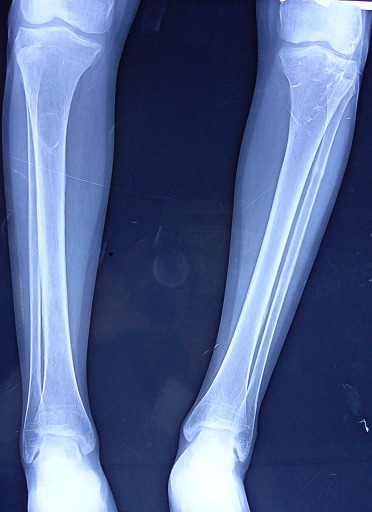
Fracture of the fibula

**Figure 5:**
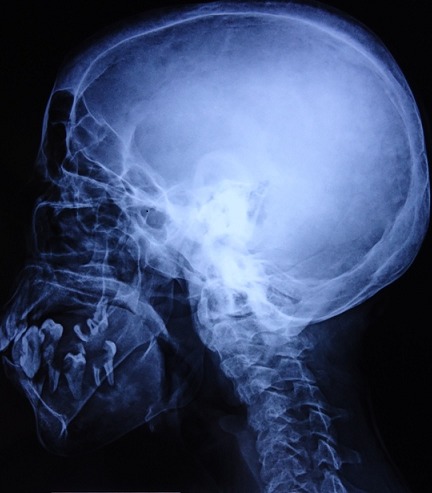
Radiography of the skull revealed a severe dental enamel defects

A 36-year-old Moroccan woman was admitted to our department because of suspected osteomalacia. Her medical history revealed growth retardation and the diagnosis of celiac disease in childhood. On admittance to our hospital, the patient complained of progressive bone and muscular pain over the last 2 years, mainly located in the ribs, spine, hips, and shoulders which had led to a double symptomatic fracture of the wrist and femur. She also had severe difficulty with walking. She had neither abdominal complaints nor diarrhea. On physical examination, she was pale. Her body mass index was 17,52. She had bad dentition, pronounced thoracic kyphosis (hyperbolic chest) with thoracic and lumbar percussion pain. Pelvis and shoulders also were painful on touching. There was muscle atrophy and symmetrical loss of proximal muscle strength. She had a painful limitation of the hips, left wrist and a genu varum (Figure 1). The dermatologic examination noted dermatitis herpetiformis at the arm (Figure 2). The relevant blood parameters were as follows: Calcemia: 92 mg/l (95-105); phosphatemia: 14 mg/l (25-45); low urine Calcium: 77,4 mg/24 h (100-300); alkaline phosphatase: 283 UI/l (35-117); low 25-hydroxy-vitamin D level <9 ng/ml (>30); PTH: 281,6 pg/ml (12-70) and iron deficiency anemia (hemoglobin: 10 g/dl, iron: 0.12 mg/l). Radiographs of the pelvis and limbs showed multiple fractures (Figure 3, Figure 4) and the radiography of the skull revealed a severe dental enamel defects (Figure 5). Bone mineral density measurement showed extremely low absolute values and T-scores: 0.617 g/cm2 (T-score:-3,9) at the femoral neck, 0.882 g/cm2 (T-score:-4,8) at the lumbar spine and 0,22g/cm2 (T-score:-5,3) at the forearm. The patient responded well clinically and biologically to a gluten-free diet, iron and calcium-vitamin D supplementation.

## Discussion

Celiac disease is a chronic digestive disease due to hypersensitivity to gliadin fraction of gluten. Classically, this disease is characterized by diarrhea, weight loss and anemia. The diagnosis is based on positive IgA and IgG antigliadin and endomysial antibodies and endoscopic detection of inflammation and atrophy of the duodenal mucosa [4]. Women comprise approximately 75% of newly diagnosed adult celiac disease and tend to have more clinically prominent disease [5]. It is often accompanied by extraintestinal complications: anemia, dermatitis herpetiformis, depression, dementia, defects of dental enamel, osteopenia or osteoporosis and osteomalacia [5, 6]. The association between celiac disease and osteomalacia has been reported for the first time in 1953 [7]. Osteomalacia was reported in some cases as indicative of celiac disease [8, 9]. Yet studies on large series of patients with celiac disease found no evidence confirming this association [3]. Osteomalacia is manifested by back pain and thighs that extend the arms and sides and a state of extreme weakness and asthenia as our patient. It has been demonstrated that celiac patients are at increased risk of fracture [10]. However, in another study, no increase in fracture risk could be demonstrated for CD [11].

The mechanism of development of bone disease in patients with untreated celiac disease is not clearly defined. Chronic hypocalcemia and vitamin D deficiency are potential factors. Bone loss is explained by the overproduction of cytokines IL-1 alpha, IL-1 beta and TNF-alpha and will be further accelerated by hyperparathyroidism secondary to malabsorption of calcium and vitamin D. these mechanisms will contribute to increased bone resorption and activate bone loss [12]. In our case, it is a severe form of osteomalacia complicating a longstanding untreated celiac disease. There were rare case reports of osteomalacia complicating chronic celiac disease [13]. This case affirms that chronic untreated celiac disease, can lead to irreversible complications like skeletal deformities and short stature.

## Conclusion

Finally, in celiac disease, osteomalacia should be suspected in order to start treatment in time to avoid the complications of bone loss.
